# The Heating and Ventilation of Hospital Wards

**Published:** 1912-12-07

**Authors:** 


### HOSPITAL ARCHITECTURE AND CONSTRUCTION.

[Communications on this subject should be marked "Architecture" in the left-hand top corner of the envelope.]

### The Heating and Ventilation of Hospital Wards.

DR. H. FRANKLIN PARSONS, in his Local Government Board Report on Isolation Hospitals, says: The windows of a ward should have an aggregate area, including frames, of about 1 square foot to 70 cubic feet of internal capacity of the ward, or to between 5 and 6 square feet of floor area. If the window area falls much short of this proportion, it tends to make the wards look dark and gloomy; if it is much in excess, the wards are more difficult to keep warm, with the result that the ventilation is apt to be restricted.

The sills of the windows, except in the case of French windows giving access to a verandah, should be about 3 feet 6 inches above the level of the floor, and the top of the windows not more than a foot below the ceiling level. Sash windows are the most suitable for hospital purposes; they are often made with a board at the bottom to exclude direct draught when the lower sash is slightly raised, thus allowing air to enter in an upward direction between the upper and lower sashes. The upper part of the window should be furnished with a hopper light, falling inwards and having side cheeks to prevent down draughts. In the Austral system (see THE HOSPITAL, April 13, 1912), which has been adopted at Altrincham Isolation Hospital, the window below the transom consists of two frames balanced together which turn on horizontal pivots and can be inclined at any desired angle.

In addition to the windows, fresh-air inlets under the beds, or Tobin's tubes, are often provided, and sometimes extracting shafts and cowls on the roofs. Ventilating stoves admitting warmed fresh air are also often used.

The warming of hospital wards is a matter requiring careful consideration. Ample means of warming are necessary where the hospital is in an elevated or exposed position, or the walls are thin, or where the wards have much outside wall in proportion to their capacity, or a large area of glass. It is right, however, to add that by some hospital physicians the warming of hospital wards is considered a matter of minor importance as compared with an abundant supply of fresh air. It is maintained that patients even with acute diseases may advantageously be treated as nearly as possible in the open air with merely the protection in severe weather of a verandah or shelter open in front, or in wards with the windows widely open; and that a low external temperature is of no consequence if the patient is kept warm by a sufficiency of clothing, with the aid, if necessary, of hot-water bottles. This is the generally accepted method of treatment of cases of pulmonary tuberculosis, and it has been in a measure adopted in the case of the acute infectious diseases, as at Nottingham. It has not, however, been proved that for all classes of cases the maintenance of the air of the wards at a reasonably comfortable and equable temperature is a matter which can be safely regarded as unimportant.

In small wards (about 24 feet by 15 feet) an ordinary fireplace is sufficient, but in larger wards (say, 36 feet by 26 feet) the question will arise of the relative advantage of heating by stoves or by a system of hot-water or steam pipes; and, in the case of a pipe system, whether the heating should be from a central boiler or from a separate boiler for each ward block. The answer to these questions will depend in a measure upon the arrangements and use of the hospital and on the amount of heating required. If the buildings are compactly arranged the heating can be effected with a less expenditure of fuel and labour from a central boiler than from a number of fireplaces, especially if the elevation of temperature to be effected is a relatively large one, as on a cold site. On the other hand, if the ward blocks are small and scattered, or if the hospital is only rarely in full use, as in the case of a small-pox hospital, the saving in current expenditure effected by heating from a central boiler may not counterbalance the interest on the capital cost of a central heating system. But even when ward blocks are not in use they require to be "aired" from time to time in order to keep them dry and fit for occupation; and a central heating system is found, as at Stroud, to be very useful for this purpose, and also to prevent freezing and bursting of pipes.

There are hygienic objections to the use of a hot-water system as the sole means of heating wards. It lacks the cheerfulness of a fireplace and, unlike the latter, does nothing directly to promote ventilation. Moreover, with a hot-water system the heating is effected in a different way, and one less agreeable to the sensations of the occupants than with fireplaces. The heating from an open fireplace takes place chiefly by radiation; the heat rays from the fire pass through the air without heating it directly, but they warm the walls and solid substances upon which they impinge, and the air is warmed indirectly by contact with these; hence the walls, floors, and furniture, etc., are warmer than the air. On the other hand, with a low-pressure hot-water system the ward is heated chiefly by convection; the layer of air next the pipes gets warmed and rises; its place is taken by a new volume of air, which is warmed in its turn; thus the air of the ward is warmed more than the walls, floor, or furniture. But when warm air impinges upon a cold surface, any solid particles suspended in the air become deposited upon the colder body, and hence in rooms heated by warm air the surfaces of walls and furniture tend to get covered with dust and dirt sooner than in one heated by radiant heat from stoves. Proximity to the colder surfaces also gives a sensation of chilliness owing to loss of heat from the skin by radiation.

Again, when air is heated its capacity for moisture is increased, and hence with a system of warming which heats the air by convection rather than the walls by radiation, the air having its degree of saturation lowered is liable to become unpleasantly dry. Methods of heating by pipes at a higher temperature, as by steam or hot water under high pressure, give off more heat in the radiant form than low-pressure hot water, but are liable to be accompanied by an unpleasant smell from heated organic matter in dust. But while there are objections to a system of heating by pipes as the sole means of warming wards, such a system may be usefully employed in addition to fireplaces in large wards or where, for reasons such as have been mentioned, ample means of heating are required. In such a case the number of fireplaces may be reduced, a single fireplace, or in long wards one in each end wall, may suffice for the purpose of cheerfulness, ventilation, and affording radiant heat, and a central stove may be dispensed with.

Central stoves, however, have a cheerful appearance, and are used in many hospitals. For large wards they are made with double fronts, so that one or both fireplaces may be used according to the requirements of the weather. They are often made, as in Shorland's and Musgrave's stoves, with descending flues, which pass under the floor to a chimney at the side of the ward. This arrangement has the advantage, as compared with a central upright flue, that there is no obstruction to the view across the ward; but it is more costly and apt to go wrong. At many hospitals where such stoves are used, complaints are made that in certain states of the weather smoke from them issues into the wards. To avoid this the effective height of the chimney above the level of the fireplace should be at least twice the length of the horizontal portion of the flue; but this involves a tall chimney in a side wall. Another objection to a flue under the floor is that the heat from it is liable to cause cracking and shrinking of the floor. Upright flues are now made of pottery, which are less unsightly than an iron pipe or brick stack, and afford little obstruction to the view across the ward; but in the lower wards of two-storey buildings stoves with descending flues are preferable, and generally act well, a high chimney being obtainable.

